# First Things First: Effectiveness and Scalability of a Basic Prehospital Trauma Care Program for Lay First-Responders in Kampala, Uganda

**DOI:** 10.1371/journal.pone.0006955

**Published:** 2009-09-11

**Authors:** Sudha Jayaraman, Jacqueline R. Mabweijano, Michael S. Lipnick, Nolan Caldwell, Justin Miyamoto, Robert Wangoda, Cephas Mijumbi, Renee Hsia, Rochelle Dicker, Doruk Ozgediz

**Affiliations:** 1 Department of Surgery, University of California San Francisco, San Francisco, California, United States of America; 2 Department of Accident and Emergency, Mulago Hospital and Makerere University, Kampala, Uganda; 3 Department of Medicine, Brigham and Women's Hospital, Boston, Massachusetts, United States of America; 4 School of Medicine, University of California San Francisco, Office of Medical Education, San Francisco, California, United States of America; 5 Department of Anaesthesia, Mulago Hospital and Makerere University, Kampala, Uganda; 6 Department of Emergency Medicine, University of California San Francisco, San Francisco, California, United States of America; 7 Department of Surgery, San Francisco General Hospital, San Francisco, California, United States of America; 8 Division of Pediatric Surgery, Hospital for Sick Children, University of Toronto, Toronto, Ontario, Canada; University of Oxford, United Kingdom

## Abstract

**Background:**

We previously showed that in the absence of a formal emergency system, lay people face a heavy burden of injuries in Kampala, Uganda, and we demonstrated the feasibility of a basic prehospital trauma course for lay people. This study tests the effectiveness of this course and estimates the costs and cost-effectiveness of scaling up this training.

**Methods and Findings:**

For six months, we prospectively followed 307 trainees (police, taxi drivers, and community leaders) who completed a one-day basic prehospital trauma care program in 2008. Cross-sectional surveys and fund of knowledge tests were used to measure their frequency of skill and supply use, reasons for not providing aid, perceived utility of the course and kit, confidence in using skills, and knowledge of first-aid. We then estimated the cost-effectiveness of scaling up the program.

At six months, 188 (62%) of the trainees were followed up. Their knowledge retention remained high or increased. The mean correct score on a basic fund of knowledge test was 92%, up from 86% after initial training (n = 146 pairs, p = 0.0016). 97% of participants had used at least one skill from the course: most commonly haemorrhage control, recovery position and lifting/moving and 96% had used at least one first-aid item. Lack of knowledge was less of a barrier and trainees were significantly more confident in providing first-aid. Based on cost estimates from the World Health Organization, local injury data, and modelling from previous studies, the projected cost of scaling up this program was $0.12 per capita or $25–75 per life year saved. Key limitations of the study include small sample size, possible reporter bias, preliminary local validation of study instruments, and an indirect estimate of mortality reduction.

**Conclusions:**

Lay first-responders effectively retained knowledge on prehospital trauma care and confidently used their first-aid skills and supplies for at least six months. The costs of scaling up this intervention to cover Kampala are very modest. This may be a cost-effective first step toward developing formal emergency services in Uganda other resource-constrained settings. Further research is needed in this critical area of trauma care in low-income countries.

## Introduction

Low and middle income countries disproportionately bear the global burden of injury and annually, an estimated one to two million injury deaths in these settings are potentially avertable through improvements in trauma care [1], [2], [3]. However, many low and middle income countries do not have formal emergency systems in place; thus an estimated 80% of injury deaths in these countries occur in the pre-hospital setting [1].

Uganda is no different. The lack of access to emergency medical care for seriously injured people in Uganda's capital city, Kampala, has been documented by others [4], [5], [6]. Since the city has no formal emergency system, pre-hospital care in Kampala is currently delivered by police, taxi drivers or community leaders (Local Council officials) [5].

To address this issue in Kampala, in 2008, we conducted a pilot study on the burden of injury seen by lay people and designed and implemented a context-appropriate course on prehospital trauma care for lay first-responders based on the World Health Organization's recommendations that lay first-responders should be the first step towards developing formal emergency medical services in settings without a formal prehospital system [7], [8], [9].

That pilot study demonstrated that lay people in Kampala encountered a substantial number of emergencies and deaths and provided emergency care in many cases but were not well-supported in this role. We also demonstrated the feasibility of designing and implementing a context-appropriate basic prehospital trauma course that increased the fund of knowledge of these lay people in essential trauma care [10]. We now report the effectiveness of this pilot program in Kampala, Uganda and estimate the costs of scaling up the program to provide emergency services across Kampala.

Since the initial training, we prospectively followed this cohort to assess the effectiveness of our training program at six months. We hypothesized that participants would retain knowledge of first-aid learned during the training course, use the skills and kit frequently and feel more empowered to provide emergency services. We also measured the costs and cost-effectiveness of scaling up this program to cover the city of Kampala based on the methods outlined in the Disease Control Priorities Project, 2nd edition (DCP2) [9].

## Methods

### First-Aid Program

Our training program, previously described in detail [10], was conducted in Kampala (population: 1.2 million) [11] and consisted of a day-long modified basic first-aid course on trauma and a free basic first-aid kit for each participant. Participants had been selected through convenience sampling from a 20 square mile catchment area around Mulago Hospital. The course curriculum was adapted from prior models by local stakeholders. The core curriculum included universal precautions, scene management, external compression for haemorrhage control, basic airway control, recovery position, safe lifting and transportation of injured victims, splinting fractures and triage. After the course, each participant received a first-aid kit assembled using locally available materials and instructions on restocking the kit.

### Study Design and Data Collection Methods

Cross-sectional surveys and fund of knowledge tests were conducted immediately before and after training and at three and six months. We collected data on the frequency and types of aid provided, supply use, barriers to providing aid, perceived utility of the course and kit and self-reported confidence in giving first-aid. Fund of knowledge tests consisted of five questions covering each core skill area. Data collection instruments were designed in English and Luganda and pilot-tested twice in Kampala before use by study personnel. Structured one-on-one interviews were used at study onset. Self-administered questionnaires were used at three months and mobile phone surveys at six months for logistical reasons. A half-day refresher session was conducted at three months.

**Figure 1:**
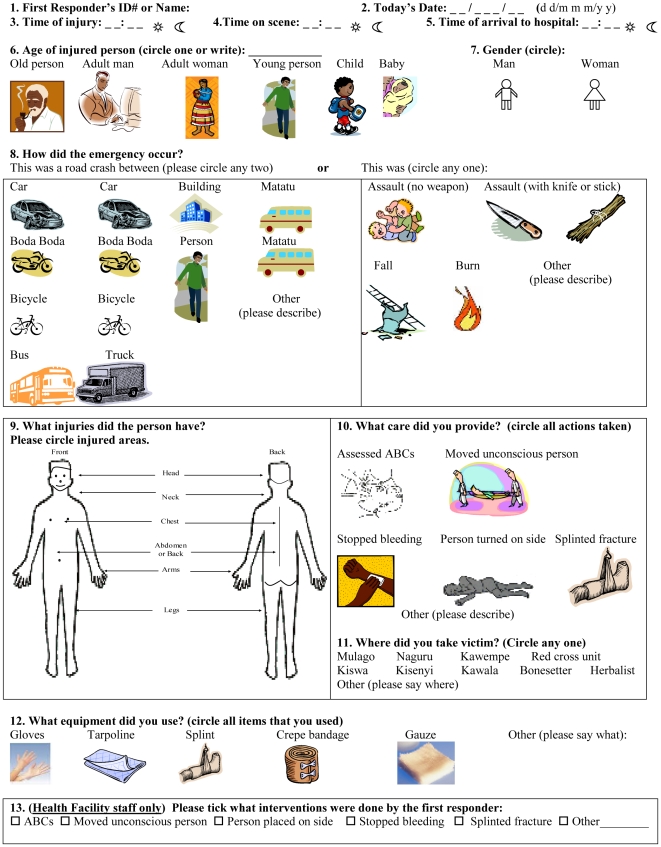
Prospective Data Collection Form for First Responders.

All trainees were also asked to complete a pictographic encounter record after each emergency they encountered during the study period. This record was designed and tested in both languages and was distributed at study onset (Figure 1). Completed records were collected at Mulago Hospital and at the five community clinics within the catchment area.

Institutional Review Board approvals were obtained from the University of California, San Francisco, Mulago Hospital and the Uganda National Council of Science and Technology. Informed consent to participate in the study was obtained from all trainees. Throughout this study, participants received no financial incentive to perform first aid in the field, or to complete encounter records. Nominal stipends were given on training days to replace lost income.

### Statistical Analysis

All data were analyzed using Stata version 10 (College Station, TX, 2007). Paired t-tests, χ2 tests and logistic regression analysis were used to compare changes in fund of knowledge, barriers to providing aid, perceived utility of the kit and course and confidence in providing aid. Spearman's correlation coefficient was used for comparing total test scores and confidence. Sub-group analysis, to compare police trainees against all others, was planned a priori, based on previous findings [9].

### Cost and Cost-Effectiveness Analysis

We measured the costs per capita, per death averted, and per life year saved of scaling up our training program based on previous projections that 6000 to 12000 lay first-responders would be needed to provide basic prehospital trauma care for Kampala's 1.2 million people. These projections, based on a conflict-affected setting, are outlined in the DCP2 [9]. We estimated projected cost-effectiveness based on recent reports on the impact of using trained lay first-responders on injury mortality [12]. Husum et al. reported a 15% mortality reduction (95% CI 8–22%) when trained lay first-responders and paramedics provided prehospital care in Cambodia and northern Iraq. Since similar estimates are not available for Kampala, we applied Husum's figures to approximately 1600 injury deaths recorded by the Mulago Hospital and Kampala City Mortuaries every year. We used these figures as the base-case scenario in Kampala and conducted deterministic sensitivity analysis to account for variations in individual cost parameters such as the impact of providing of first-aid supplies and a more conservative 7.5% mortality reduction on costs per death averted and LY saved.

## Results

### Effectiveness of First-aid Program

We enrolled 309 trainees in the study and were able to evaluate 127 participants (41%) at three months and 188 (62%) at six months.

#### Perception of training program and ability to provide first-aid

Trainees rated the course and kit highly throughout the six months. Overall, on a scale of one to five, with five being extremely useful, 98% of trainees rated the course at 4 or 5 out of 5 at three months (n = 124) and 99% rated it the same at six months (n = 185). On the same scale, at three months, 97% rated the kit 4 or 5 out of 5 (n = 125) and 94% did the same at 6 months (n = 185). When trainees' confidence in providing first-aid for trauma was measured from zero to five, the mean before the training course was 3.1. At three months, this increased to 4.2 (n = 124, p<0.0001) and remained at that level at six months (n = 181, p<0.0001). There was no correlation between increasing levels of confidence and mean test scores, at six months.

#### Self-reported Use of First-Aid Skills and Supplies

At three months, 84% of participants had used at least one skill taught in the course. By six months, this frequency increased to 97%. At six months, the most commonly used skills were haemorrhage control, recovery position and lifting/moving (table 1). In paired analysis, participants reported using basic airway control and external compression for haemorrhage control significantly more frequently at six months than they did at before the course but used safe lifting and moving techniques significantly less frequently (table 2).

**Table 1:** Most commonly used skills and supplies from the first-aid course.

Rank	Survey data n = 179	Encounter records n = 153
*Skills*		
1	Hemorrhage control (74%)	Lifting/moving (47%)
2	Recovery position (65%)	Hemorrhage control (42%)
3	Lifting/moving (65%)	Recovery position (40%)
*Supplies*		
1	Gloves (92%)	Gloves (83%)
2	Cotton (79%)	Gauze (63%)
3	Gauze (74%)	Bandage (43%)

**Table 2:** Use of specific skills before and six months after the first-aid course.

Skill	Before Training	Six Months After Training*	P value†
Checking airway/breathing	50%	64%	0.0041
Control bleeding	61%	74%	0.0075
Splinting	31%	35%	NS
Lifting/moving	78%	67%	0.0072
Recovery position‡	43%	57%	0.04

* n = 171 pairs, except for checking airway/breathing: n = 169 pairs.

† by paired t test.

‡ was evaluated at three months and not at initial training.

At three months, 85% of participants had used at least one component of the study kit and by six months, this frequency increased to 96%. At six months, the most commonly used items were gloves, cotton and gauze (table 1). Every item was considered “hard to stock” by trainees at 6 months despite the restocking help provided by the study team.

#### Barriers to Providing First-Aid

Inability to provide aid due to lack of knowledge decreased significantly at six months compared to pre-training although more trainees were unable to help in an emergency due to work or travel-related reasons (table 3).

**Table 3:** Reported reasons for not providing assistance.

Reason*	Before Training	At six months	P value†
I did not know what medical help was needed	8%	3%	0.018
I did not feel safe providing help	2.8%	3.3%	NS
I did not have equipment to provide help	6%	9.5%	NS
I could not help due to work-related reasons	2.8%	13%	0.0003
I could not help because I was travelling and could not stop	2%	0.12%	0.0006
I had other reason for not providing aid (n = 178)	4.5%	8.4%	NS
I had more than one reason to not provide aid (n = 180)	6%	9%	NS

* n = 179 unless otherwise noted

† by paired t test

#### Knowledge Retention

At six months, trainees answered 92% of all fund of knowledge questions correctly (mean %) compared to 86% immediately after the initial training (n = 146 pairs, p = 0.0016).

Over 95% of trainees scored correctly on scene management, basic airway control and recovery position and 80% did so on external compression for haemorrhage control and safe transportation (table 4). Attending the three month refresher training did not affect the fund of knowledge test scores at 6 months.

**Table 4:** Participants' fund of knowledge at six months compared to after initial training.

	% answering correctly immediately *after* training	% answering correctly at six months	p value*
Scene management (n = 146)	95%	98%	NS
Checking airway/breathing(n = 146)	92%	95%	NS
External compression for haemorrhage control (n = 147)	76%	84%	0.04
Use of recovery position (n = 145)	94%	95%	NS
Speeding to hospital (n = 138)	87%	80%	NS

* by paired t test.

### Sub-group analysis

#### Effect of being a Police trainee on outcomes at six months

Police trainees were likely to have a higher test score at 6 months compared to other trainees and this persisted after controlling for refresher training attendance (OR = 2.3, p = 0.010). They were also more likely to report using the recovery position (OR = 1.9, p = 0.05), safe lifting or moving techniques (OR = 2.8, p = 0.002) and splinting (OR = 1.9, p = 0.06) at six months, than the other trainees. These findings were adjusted for refresher course attendance. When frequencies of skill and supply use were measured, police trainees reported using both more frequently than other trainees (skill use: OR = 1.9, p<0.025 and kit use: OR 3.1, p<0.0001).

#### Correlation of survey data with encounter records

We collected 153 encounter records prospectively from trainees over the six month study period. Police officers submitted 44% of records, followed by Local Council officials (24%) and taxi drivers (16%). Injured victims were mostly young men (median age = 25, male = 88%) with injuries most commonly to the extremities (67%) or head and neck (52%). Skill and supply use reported were similar to findings from the cross-sectional surveys (table 2). Time from injury to response by study participants and subsequent arrival to the hospital was 39 min (median). In comparison, the Mulago Hospital Trauma Registry data notes a median of 120 minutes from time of injury-to-hospital arrival.

### Cost Analysis

The WHO and World Bank suggest that 9000 trainees (range 6000–12000) would need to be trained to provide prehospital services for Kampala's 1.2 million people. For a base case scenario, we calculated the annual cost of training 9000 trainees over 3 years using our program. This resulted in a projected annual cost of $47,854 and a per capita cost of $0.12 for the base case (table 5). When one-way sensitivity analysis was to evaluate the addition of a $16 first-aid kit and an additional $16 restocking supplies, we found that overall costs ranged up $143,854 and per capita costs up to $0.36 (table 6).

**Table 5:** Annual cost of training 3000 lay first-responders (Base case).

Trainees for full-day training	3000
Trainee salary at WHO level 1	$14,610
Trainer days at 6 trainers & 50 trainees per day	360
Trainer salary at WHO level 3	$3,244
Room rental ($75/day)	$27,000
Materials ($1/trainee)	$3,000
**Total costs for base case**	**$47,854**

**Table 6:** Costs per capita of training 9000 lay first-responders for Kampala.

	Annual Costs	Costs to train all 9000	Costs per capita
Base case	$47,854	$143,561	$0.12
Base case with $8 kit	$71,854	$215,561	$0.18
Base case with $8 kit and $8 worth of extra supplies	$95,854	$287,561	$0.24
Base case with $16 kit	$95,854	$287,561	$0.24
Base case with $16 and $16 worth of extra supplies	$143,854	$431,561	$0.36

### Projected Cost-Effectiveness of Implementing Lay First-Responder Training in Kampala

If our program was scaled up to cover Kampala, 240 deaths (15%) could be averted every year, which results in a projected cost per death averted of $598 using the base case or a cost per life year (LY) saved of $25 based on an average age of death from injury of 27 with a life expectancy of 24 additional years at that time. Results of the sensitivity analysis when a $16 kit and supplies were included showed that costs per death averted increased to a maximum of $1798 or $75 per life year saved. When the more conservative (7.5%) mortality reduction was used, this upper limit further increased to $3596 per death averted or $150 per life year saved (table 7).

**Table 7:** Cost per death averted and per life year saved of training 9000 lay first responders.

Estimated Deaths averted, n (%)	240 (15%)120 (7.5%)
	Costs to train all 9000	Cost per death averted	Cost per LY saved	Cost per death averted	Cost per LY saved
Base case	$143,561	$598	$25	$1,196	$50
Base case with $8 kit	$215,561	$898	$37	$1,796	$75
Base case with $8 kit and $8 worth of extra supplies	$287,561	$1,198	$50	$2,396	$100
Base case with $16 kit	$287,561	$1,198	$50	$2,396	$100
Base case with $16 and $16 worth of extra supplies	$431,561	$1,798	$75	$3,596	$150

## Discussion

To address global disparities in trauma care, the World Health Organization (WHO) Essential Trauma Care Guidelines outline the “needs of the injured patient,” such as airway management and bleeding control, as essential health services for all people regardless of resources or context. The mandate of health systems to provide essential services has also been stressed through the rights-based approach to health [13]. The availability of adequate emergency medical services is often considered a basic human right in high-income countries [14], [15], [16] and this right should not be neglected in low and middle income countries which bear the disproportionate burden of injury. In addition, calls for injury research in resource-constrained settings have particularly stressed the need for intervention-based research [17].

Our study thus fills these critical needs. Despite a substantial burden of injury in Uganda, Kampala, its largest city, has no formal prehospital emergency system. We found that lay people can effectively retain knowledge of prehospital trauma care learned through a context-appropriate first-aid course for at least six months. Trainees found this basic intervention useful and after the training, were able to more confidently deploy these new skills. Our findings suggest that police may be the ideal first-responders in Kampala given their higher knowledge retention and skills and supply use compared with other trainees. Their established communication and transportation networks are also an advantage. However, appropriate recognition for such services will be critical to dissuade any incentive to charge informal fees for services. This study also showed shorter times from injury to hospital arrival, although this compares two different datasets; our prospectively collected encounter records and the hospital trauma registry. Nonetheless, the findings suggest that delivery of prehospital care did not delay access to care.

This study can be compared with others' experiences in developing prehospital systems in resource-constrained settings. In urban Ghana, truck drivers who completed a similar context-appropriate first-aid course showed a significant increase in skill use at 10 months based on response rate of 28% [18]. However, trainees assembled first-aid kits at their own expense and at 10 months, only 27% of trainees carried materials useful for universal precautions such as gloves or plastic bags. Our study conducted more in-depth and periodic assessments of participants' fund of knowledge and use of free first-aid kits.

Husum et al. have demonstrated a 15% reduction in mortality of war and landmine victims in Iraq and Cambodia where paramedics and lay first-responders were trained to provide prehospital trauma care [12], [19], [20]. Despite extensive training programs for both groups, most prehospital care provided was solely basic life support. Others have argued for implementation of this model in non-conflict settings without formal emergency systems [9]. We designed our study based on a belief that a simple “scoop-and-run” approach with basic life-saving interventions will be most practical and effective in the absence of second-tier responders such as paramedics who are trained in advance airway management, vascular access and fluid resuscitation. In addition, we could not provide extensive training for our participants due to opportunity costs of removing police officers from duty and imposing a greater loss of income for the taxi drivers. Therefore, while not directly comparable to previous work, our study suggests that high knowledge retention and greater use of first-aid skills may be rough proxies for improvements in trauma care.

We also estimated that the costs of scaling up this intervention to cover all of Kampala's 1.2 million people was $25–150 per life year saved ($598–$3596 per death averted) which is comparable to the costs of traffic enforcement in Kampala at $27 per life year saved [21] or the distribution of antiretroviral medications for HIV in Uganda at $600 per life year saved [22]. As such, this intervention could be considered very cost-effective. Additionally, Uganda's health policy is based on a minimum package of essential health services, which was initially costed at $34 per capita by the WHO [23], [24]. Current public expenditure on health is USD $22 per capita in Uganda [25]. Thus, $0.12 per capita would be a modest price to pay for prehospital care, especially since the current package does not include emergency services for trauma.

However, our study has several limitations. First, we did not directly measure changes in mortality. Our small sample size and poor health system infrastructure limit the capacity to follow an injured victim across the continuum of care. Second, skill and equipment use may have been over-reported by participants. Although we attempted to address this using prospective encounter records, hospital-level outcomes data would have increased the rigor of our study. Third, our study design may not be ideal. While a randomized controlled trial is the gold standard to prove intervention effectiveness, its use to test the benefits of essential skills of basic trauma care in the prehospital setting is not ethical. Furthermore, we did not use a control group in this study and relied on before-and-after comparisons to establish effectiveness which may be less than ideal.

Other contextual factors may have also affected our results. We could not control for changes in the frequency of skill use based on changes in job posting. Police trainees are rotated periodically, which removes them from active field duty, and thus potentially limits their ability to be present at emergencies. During the study, more trainees reported being unable to assist in emergencies due to work or travel related reasons suggesting that trainee selection was not ideal or that opportunity costs prohibited our trainees from responding to emergencies. Finally, the implications of some of our results remain unclear. Some skills, such as safe lifting and moving, were reported less frequently at six months than after the initial training perhaps because their new knowledge or increased credibility as emergency providers allowed them to harness help to transfer victims in emergencies or perhaps this skill simply may need more emphasis in future training.

Our cost and cost-effectiveness analyses must be interpreted with caution, even though sensitivity analyses were used. Estimates of the number of lay first-responders needed for Kampala and their potential impact on mortality were determined based on work primarily in a conflict-affected setting. By comparison, a decreased incidence of trauma and associated frequency of skill use in urban Kampala may have led to an overestimate of the cost-effectiveness.

Despite study limitations, many areas for further research exist. Better methods are needed to measure the impact of training lay first-responders on trauma morbidity and mortality. While we established the need for a simple first-aid kit for effective provision of prehospital care, mechanisms to ensure restocking of kits also need to be determined. Facility-based records could more accurately measure economic burden and may help determine the accuracy of our projections.

Training lay first-responders may be a critical and cost-effective first step to develop a formal emergency medical system. However, this intervention needs to be complemented by a second-tier of responders, a call center, and appropriate transportation mechanisms as well as facility-based training for clinicians to provide appropriate trauma care when victims reach health facilities. The precedent for such comprehensive trauma systems development is being established in other resource-constrained settings and could provide practical lessons for Ugandan policy-makers [26], [27], [28], [29], [30]. Since injury disproportionately affects the working poor who rely on high-risk modes of transport such as non-motorized vehicles, motorized two-wheeled vehicles and foot traffic, by improving access to emergency services, a formal prehospital emergency system in Uganda can become a *‘pro-poor’* strategy and increase equity [31], [32]. Additionally, organized emergency services could improve health system efficiency by building on existing informal mechanisms of care. Most importantly, the global health community must urgently provide greater priority and resources for injury intervention research in resource-constrained settings that are particularly burdened with high incidence of trauma.

In conclusion, a lay first-responder training program is a practical and effective first-step towards developing a formal emergency system in Uganda. It is likely to be very cost-effective in this setting. Establishing and scaling up this intervention with in Kampala should be a key priority for Ugandan policy-makers. Incorporating emergency services into the essential package of health care would critically address the disproportionate global injury burden shouldered by the poor. Results of this program could be useful in other resource-constrained settings that lack emergency medical systems.

## Acknowledgments

We would like to sincerely thank the following contributors who gave valuable assistance at during various stages of this project: Moses Dumba from the Ugandan Taxi Operators and Drivers Association, Red Cross First-Aid Instructors: Robert Okuyat, Juliet Kiyimba, Ivan Luwaga, Daniel Nsubuga, and Red Cross Volunteer: Yusuf Kimbowa. Sarah Nakitto, Research Coordinator. Thanks also to UCSF Department of Surgery Scientific Publications Manager, Pamela Derish for editorial support and Dr. Kayvan Roayaie for statistical assistance.
